# Hyper-Enriched Anti-RSV Immunoglobulins Nasally Administered: A Promising Approach for Respiratory Syncytial Virus Prophylaxis

**DOI:** 10.3389/fimmu.2021.683902

**Published:** 2021-06-07

**Authors:** Emilie Jacque, Claire Chottin, Daphné Laubreton, Michel Nogre, Cécile Ferret, Sandrine de Marcos, Linda Baptista, Carole Drajac, Philippe Mondon, Christophe De Romeuf, Marie-Anne Rameix-Welti, Jean-François Eléouët, Sami Chtourou, Sabine Riffault, Gérald Perret, Delphyne Descamps

**Affiliations:** ^1^ LFB Biotechnologies, Les Ulis, France; ^2^ Université Paris-Saclay, INRAE, UVSQ, VIM, Jouy-en-Josas, France; ^3^ Université Paris-Saclay, UVSQ, Inserm, Infection et inflammation, U1173, Montigny-Le-Bretonneux, France; ^4^ AP-HP, Hôpital Ambroise Paré, Laboratoire de Microbiologie, Boulogne-Billancourt, France

**Keywords:** concentration, nasal administration, bioprocess, human plasma, hyper-immune immunoglobulins, prophylactic strategy, neutralization, viral infection

## Abstract

Respiratory syncytial virus (RSV) is a public health concern that causes acute lower respiratory tract infection. So far, no vaccine candidate under development has reached the market and the only licensed product to prevent RSV infection in at-risk infants and young children is a monoclonal antibody (Synagis^®^). Polyclonal human anti-RSV hyper-immune immunoglobulins (Igs) have also been used but were superseded by Synagis^®^ owing to their low titer and large infused volume. Here we report a new drug class of immunoglobulins, derived from human non hyper-immune plasma that was generated by an innovative bioprocess, called Ig cracking, combining expertises in plasma-derived products and affinity chromatography. By using the RSV fusion protein (F protein) as ligand, the Ig cracking process provided a purified and concentrated product, designated hyper-enriched anti-RSV IgG, composed of at least 15-20% target-specific-antibodies from normal plasma. These anti-RSV Ig displayed a strong *in vitro* neutralization effect on RSV replication. Moreover, we described a novel prophylactic strategy based on local nasal administration of this unique hyper-enriched anti-RSV IgG solution using a mouse model of infection with bioluminescent RSV. Our results demonstrated that very low doses of hyper-enriched anti-RSV IgG can be administered locally to ensure rapid and efficient inhibition of virus infection. Thus, the general hyper-enriched Ig concept appeared a promising approach and might provide solutions to prevent and treat other infectious diseases.

**Importance:**

Respiratory Syncytial Virus (RSV) is the major cause of acute lower respiratory infections in children, and is also recognized as a cause of morbidity in the elderly. There are still no vaccines and no efficient antiviral therapy against this virus. Here, we described an approach of passive immunization with a new class of hyper-enriched anti-RSV immunoglobulins (Ig) manufactured from human normal plasma. This new class of immunoglobulin plasma derived product is generated by an innovative bioprocess, called Ig cracking, which requires a combination of expertise in both plasma derived products and affinity chromatography. The strong efficacy in a small volume of these hyper-enriched anti-RSV IgG to inhibit the viral infection was demonstrated using a mouse model. This new class of immunoglobulin plasma-derived products could be applied to other pathogens to address specific therapeutic needs in the field of infectious diseases or even pandemics, such as COVID-19.

## Introduction

Plasma derived Immunoglobulins (Ig) contain a wide spectrum of specific antibodies to different pathogens and are used to reduce infections in immunocompromised patients. The protective effect of polyclonal immunoglobulins administered intravenously (IVIg) is controversial and does not totally prevent the occurrence of infectious diseases. High doses of IVIg seem to significantly reduce the number and duration of infections in primary immune deficiency diseases (1). However, despite the optimal level of IgG (8 g/L) and adequate antibiotics, some patients still develop bowel infections or chronic sinusitis and bronchiectasis caused by *Haemophilus influenzae* and *Streptococcus pneumoniae* (2). In contrast, human immunoglobulins enriched in target-specific antibodies (1-5%, called hyper-immune) have been and are still being used with success in preventing viral or bacterial infections with hepatitis B virus, tetanus, Cytomegalovirus and rabies virus (3). Nevertheless, the efficient doses remain high in these specific indications and administration side effects have sometimes been reported. In parallel, the use of monoclonal antibodies (mAbs) as anti-infective products is rising (4). Indeed, the emergence of new infectious diseases, the re-emergence of ancient infectious diseases, the rise of antibacterial resistance to conventional antibacterial agents and the difficulty in introducing and supplying a new pipeline of novel antimicrobial compounds increase the interest for non-conventional approaches. However, this class of immunotherapy using mAbs remains limited by prohibitive costs and pathogen resistances related to the mono-specificity (5).

In this paper, we describe a new drug class of immunoglobulins, derived from human plasma that was generated by an innovative bioprocess to meet specific therapeutic needs in the infectious disease area. The concept, called Ig cracking, gathers expertises in both plasma-derived products and affinity chromatography. The breakthrough innovation of Ig cracking will provide a product composed of at least 15-20% antibodies specific to a viral or a bacterial target from either normal plasma or purified polyclonal immunoglobulins. This first-in-class product is called hyper-enriched immunoglobulin. The advantages of poly-clonality will be conserved, i.e. i) a higher efficacy linked to various functionalities including polyneutralization and heteroligation effects (6) and ii) the risk mitigation against escape mutations. Moreover, with at least 15-20% of specific active substance, the high dose and long infusion, which are major drawbacks of hyper-immune Igs (purity less than 1%) will be addressed. The same batch of plasma could be used to generate several hyper-enriched Igs against different antigenic targets. The depleted material could even be used to purify IVIg for inflammatory disease treatments for which, irrespective of the therapeutic mechanisms currently proposed, pathogen-specific Ig are not required.

As a first proof of concept, the respiratory syncytial virus (RSV) has been chosen. RSV is an enveloped virus that belongs to the family of *Pneumoviridae*. RSV infection has an estimated global incidence of 33 million cases in children younger than 5 years, with 10% requiring hospital admission and up to 199,000 dying of the disease (7). Recently, the Pneumonia Etiology Research for Child Health (PERCH) study demonstrated that RSV was the most common pathogen in severe pneumonia affecting young African and Asian children (8). In addition, there is growing evidence of a causal relationship between infant bronchiolitis and later asthma (9). According to recent progress in the diagnosis of RSV infection, this virus is so prevalent that it is estimated to cause a disease burden superior to influenza in the elderly and other fragile (*i.e* immunocompromised) adults (10, 11). Given the aging population structure in Western countries, adult RSV-associated mortality is becoming a huge health and economic burden, even greater than RSV-associated bronchiolitis in infants (12). In contrast, in healthy immunocompetent adults, RSV infection is controlled and ranges from symptom-free to a mild cold (13). Our aim is to transfer natural molecules able to control RSV infection from healthy adults to fragile patients, like those with impaired mucosal immunity (Chronic Obstructive Pulmonary Disease (COPD), lung transplant or Cystic fibrosis patients), in a highly purified and concentrated form. These hyper-enriched anti-RSV Ig were purified using as affinity ligand a potent antigen for virus-neutralization, the RSV fusion protein (F protein). This surface glycoprotein is more conserved than the other surface glycoprotein G (89% versus 53% amino acid sequence identity respectively for these proteins of RSV subgroup A compared with subgroup B) and plays an essential role in infection (14, 15). The F protein can take two structural conformations, the post-fusion form (postF) which is very stable (16) and the pre-fusion form (preF) which is metastable unless stabilizing mutations are introduced (17). Both structural conformations display neutralizing epitopes, some shared between the pre- and the post-fusion forms (sites I, II and IV) (18). Site II is the neutralizing epitope targeted by the monoclonal antibody palivizumab (Synagis^®^). Profiling of RSV antibody repertoire in adults shows that a relatively large proportion of the antibodies binds exclusively to preF and that the vast majority of remaining antibodies binds to both pre- and postF, with highly potent neutralizing antibodies being specific for preF (19).

We describe for the first time, the purification of hyper-enriched anti-RSV IgG manufactured from normal (non hyper-immune) plasma using the extracellular domain of F (RSV-A2 strain, Met1-Thr529) as affinity ligand for RSV-specific Ig. The strong efficacy of this hyper-enriched product was first shown *in vitro* and secondly in an *in vivo* model of infection adapted from the previously described bioluminescent RSV model (20). Our results demonstrated the high potential of this novel hyper-enriched Ig product anti-RSV that can be administered locally to ensure rapid and efficient inhibition of virus infection and transmission.

## Materials and Methods

### Human Plasma

Plasma used for the preparation of the anti RSV antibodies is what is called normal plasma by opposition to hyperimmune plasma obtained from repeatedly immunized donors. Plasma is obtained either from whole blood donors or plasma donors. Human normal immunoglobulin for intravenous administration (IVIg) provided by LFB Biotechnologies (France) and used as starting material has been produced by fractionation of pooled human plasma from 20,000 to 40,000 healthy donors for each batch and thus contain a wide range of IgG reactivity (30). In term of geography, plasma is collected in Europe (mostly in France). Donor’s age is ranging from 18 to 65 years. Donors are not selected according to their serology or history with RSV infection. They are deemed to be representative of the adult population in EU countries in terms of seropositivity and anti-RSV titers. Thus, initial concentration of anti-RSV Ig reflects the overall history of prior RSV infection from these numerous donors. The use of normal plasma pools to extract, concentrate and make available to patients these hyperimmune specific immunoglobulins is our policy choice since hyperimmune plasma is increasingly rare and difficult to obtain, unlike normal plasma whose current volume exceeds 60 million liters per year worldwide.

### F-RSV Affinity Chromatography

GE Healthcare’s NHS-activated Sepharose™4 Fast Flow was used for ligand immobilization of RSV fusion F protein (F-11049 V08B, Sino Biological Inc., https://www.sinobiological.com/recombinant-proteins/rsv-rsv-fusion-11049-v08b) and all specific anti-RSV IgG chromatography experiments. Preparative scale batch was carried out using Äkta Avant instrument (GE Healthcare, Uppsala, Sweden) controlled by Unicorn 7.0 software. Polyclonal IgG solution (ClairYg^®^ Intermediate IVIg Product, LFB, France) containing 0.11% for specific anti-RSV IgG content, was used as source material. Intermediate IVIg product process included ethanol fractionation of the plasma pool obtained from blood donation, caprylic acid precipitation to remove impurities, depth filtration and activated carbon adsorption before anion exchange chromatography for IgA and IgM removal. The eluate was thoroughly diafiltered in water and stored in liquid state at + 2/8°C until used for F-RSV affinity chromatography. Optimal capture was previously determined by Design of experiments (GE Helthcare Life Sciences): 0.7 g F protein (F-11049 V08B, Sino Biological Inc.) per L of gel – 5 minutes, the resident time needed for eluting component to pass through the column (1% IgG final concentration) in 10 mM trisodium citrate containing 0.05 M sodium chloride and pH adjusted to 7.4 with 1 M Tris solution allowed 80% of specific antibodies capture. After loading, the column was subjected to an extensive wash with the equilibration buffer until baseline return before elution at pH 2.5 with 100 mM Glycine-HCl buffer in the presence of 30% propylene glycol. The matrix was regenerated after each cycle using 6 M guanidine-HCl buffer. About 60 mg of specific anti-RSV IgG was recovered from 12 cycles using an 11-mL column of F-RSF affinity matrix. Following elution, the pH was neutralized and the pool of eluates was subjected to a polishing step on 1-mL Sartobind-Q nano capsule (Sartorius-Stedim Biotech) in order to reduce potential bacterial endotoxins and aggregates. The flow-through fraction was then concentrated to 1.5 g/L using a Biomax™ 50 kDa membrane (Pellicon™ XL, Merck-Millipore) and dialysed against ClairYg^®^ formulation buffer (vehicle) containing Mannitol and Glycine as excipients for IgG stabilization before a final 0.2 µm filtration (Optiscale™, Merck-Millipore), filling in sterile flasks and freezing at < – 60°C. The integrity of the hyper-enriched anti-RSV immunoglobulins was characterized by Coomassie blue staining SDS PAGE and compared to a commercial normal polyclonal IgG (ClairYg ^®^).

### Characterization of Enrichment Process

The total IgG of each anti-RSV Ig batch was determined by nephelometric assay (BN™ II, Siemens Healthcare). The preparation was titrated for RSV-specific IgG against recombinant human RSV F protein from different viral strains (Strain Long and Strain RSS-2 from Sino Biological Inc, 40039-V08B and 40037-V08B, respectively) using by Mesoscale Discovery™ (MSD) Multi-Array technology (Meso Scale Diagnostic) with the standard IgG Synagis (100 mg/mL, 20002783, AbbVie).

### Neutralization Tests

Anti-RSV Ig antiviral activity was assessed by measuring its ability to inhibit RSV infection of human epithelial cells, HEp-2 cells (clone CCL-23, ATCC), cultivated in MEM containing 5% FCS, 1% glutamine and 1% antibiotics (100 U/mL penicillin and 100 μg/mL streptomycin, Invitrogen). Cell infection assays were performed with recombinant RSV that was derived from the Long strain to express mCherry red fluorescent protein (RSV-cherry) (20). MEM medium without phenol red was used for cell culture and virus dilutions. HEp-2 cells were seeded at 5 × 10^4^ cells in 100 µL of MEM medium containing 5% FCS for one day. Equal volumes of diluted virus suspension were mixed with antibody dilutions (from 1:2.5 to 1:3814). After 1 h incubation at 37°C, in 7% CO_2_, virus-antibody mixtures were transferred to cell monolayers. Each sample dilution was run in triplicate with the appropriate positive control (anti-RSV-F Ig Synagis™ 100 mg/mL, 20002783, AbbVie). After 36-48 h incubation at 37°C in 7% CO_2_, cell infection was assessed by measuring mCherry fluorescence (expressed in relative fluorescence units) on a Tecan infinite M200PRO spectrofluorometer with excitation and emission wavelengths of 580 and 620 nm, respectively. Non-infected HEp 2 cells were used as standards for fluorescence background levels; 100% infection controls consisted in HEp-2 cells incubated with diluted virus suspension only (no antibodies). For each sample, % RSV infection was plotted against Ig logarithmic concentration as a dose-response inhibition of HEp-2 infection. Neutralisation of a clinical RSV isolate was performed by flow cytometry. In brief, RSV clinical strain (strain n°666649 obtained by CNR Caen de la Rougeole et des Paramyxoviridae respiratoires, France) was incubated with serial dilutions of anti-RSV antibodies (Synagis™ or anti-RSV Ig). After one hour at 37°C, HEp-2 cells were added for 48 hours then dissociated using Tryple Select enzyme solution. After washing in RPMI + 5% FCS, RSV-infected cells were determined by flow cytometry using rabbit anti RSV-F (Sino Biologicals, 11049-R09) and secondary anti-rabbit PE (Cell Lab, 732751) to determine expression of F protein at the HEp-2 cell surface. Results were expressed in MFI, 100 arbitrarily being the MFI obtained with Hep-2 infected cells without anti-RSV antibodies.

### Mice

Female BALB/c mice aged 7-8 weeks were purchased from Janvier (Le Genest, St. Isle, France) and housed under Bio-Safety Level-2 conditions (IERP, INRAE, Jouy-en-Josas, authorization number 15-50). All experiments were approved by the local ethics committee COMETHEA (INRAE and AgroParisTech) and were performed according to the European Community rules of animal care.

### *In Vivo* Administration and Viral Infection

Mice received anti-RSV Ig by the intraperitoneal (I.P.) route (0.1, 1 or 10 mg/kg in 235 μL, *n* = 4) or by the intranasal (I.N.) route (0.1 or 1 mg/kg in 50 μL, *n* = 4) after anesthesia with ketamine and xylazine (50 and 10 mg/kg, respectively, I.P. route). As positive controls of viral infection, mice were treated with ClairYg^®^ formulation buffer (vehicle) or irrelevant antibodies (control Ig or CTL Ig). Twenty hours after the treatment, mice were anesthetized and infected I.N. with a recombinant RSV expressing luciferase derived from the Long strain (RSV-Luc, 7 × 10^5^ pfu/mice in 50 µL) (20). At 4 day post-infection (d.p.i.), mice were euthanized with pentobarbital injection (I.P. route, 150 µL/mice). The lungs were collected and kept frozen at –80°C until processed for quantification of luciferase activity.

For studies to determine local protection of the nasopharynx, anti-RSV Ig (0.01 to 1 mg/kg) were intranasally instilled in a small volume (10 µL) to limit the diffusion of antibody (Ab) from the upper respiratory tract. One or 5 hours after treatment, mice were infected I.N. with 1.4 × 10^5^ pfu RSV in 10 µL in order to follow viral replication mainly in nasopharynx (upper respiratory infection model). At 1 or 4 d.p.i., mice were euthanized, and the lungs and nasal turbinates (NT) were collected and kept frozen at –80°C until processed for quantification of luciferase activity.

### Determination of RSV Viral Load in Living Mice by Bioluminescence

Viral loads were measured in living mice *via* photon emission that was representative of RSV-Luc replication using the IVIS system (Xenogen Biosciences), as previously described (20). Briefly, mice were anesthetized by intramuscular injection of ketamine + xylazine (50 and 100 mg/kg, respectively). Then, all animals received 50 µL of D-luciferin substrate (30 mg/mL, Perkin Elmer) I.N. and placed in the IVIS-200 system light detector (Xenogen Advanced Molecular Vision). Bioluminescence images were acquired using Living Imaging software for 1 min with f/stop = 1 and binning = 8. A digital false-color photon emission image of the mouse was generated. Luciferase expression was determined by measuring the number of photons emitted from the dorsal view of mice per second and was expressed by radiance (photons/sec/cm^2^/sr).

### Measure of Luciferase Expression in Lung and Nasal Turbinate Homogenates

RSV-Luc replication was measured in homogenates of the lungs and NT as previously described (31). The lung or NT samples were weighed and tissues were homogenized in passive lysis buffer 1X (30 mM Tris pH 7,9; 10 mM MgCl2; 1,25% Triton X100; 18,75% glycerol) using a Precellys (Bertin). Then, 50 µL of lysate was deposited in wells of black half-area microplates and luminescence was measured using the IVIS system after addition of D-luciferin substrate (50 µL, Promega). Photon emission was counted within a constant region of interest (ROI) corresponding to the surface of each well in the microplates, and was expressed in radiance (photons/sec/cm2/sr) that was normalized to the organ weight.

### *N Viral RNA* Expression by Quantitative Real-Time (RT)-PCR

Total RNA was extracted from homogenates of the left lung lobe from each mouse using NucleoSpin RNA columns (Macherey-Nagel) according to the manufacturer’s instructions. RNA samples were reverse transcribed using random primers and M-MLV Reverse Transcriptase (SuperScript II, Invitrogene) according to the manufacturer’s instructions. The primers (from Sigma–Aldrich, St. Louis, MO) used are RSV N gene: F-5’-AGATCAACTTCTGTCATCCAGCAA-3’ and R-5’-TTCTGCACATCATAATTAGGAGTATCAAT-3’ and mHPRT gene: F-5’- CAGGCCAGACTTTGTTGGAT-3’ and R-5’-TTGCGCTCATCTTAGGCTTT-3’. Real time PCR was run in triplicate for each gene in 96 well microplates using the MasterCycler^®^ ep realplex (Eppendorf) and SYBRGreen PCR Master Mix (Eurogentec). Fluorescence curves were analyzed using the Realplex software (Eppendorf) to determine the cycle threshold (Ct) values for each gene. Individual data were normalized to HPRT mRNA, by calculating the ΔCt (median Ct (gene) - median Ct (HPRT)) and the ΔΔCt value (sample ΔCt - mean ΔCt of control group) was used to calculate relative gene expression (2^-ΔCt^) or relative quantity using the formula RQ=2^-ΔΔCt^.

### Statistical Analysis

Data were expressed as arithmetic mean ± standard error of the mean (SEM). Statistical analyses were performed using GraphPad Prism software (version 8.1.2). Nonparametric two-tailed Mann–Whitney test was used to compare unpaired values (comparison of two groups, n ≥ 4). Significance is represented on graphs: *P < 0.05; **P < 0.01; ***P < 0.001).

## Results

### Efficacy of Hyper-Enriched Anti-RSV Immunoglobulins to Neutralize Several RSV Strains

**Figure 1:**
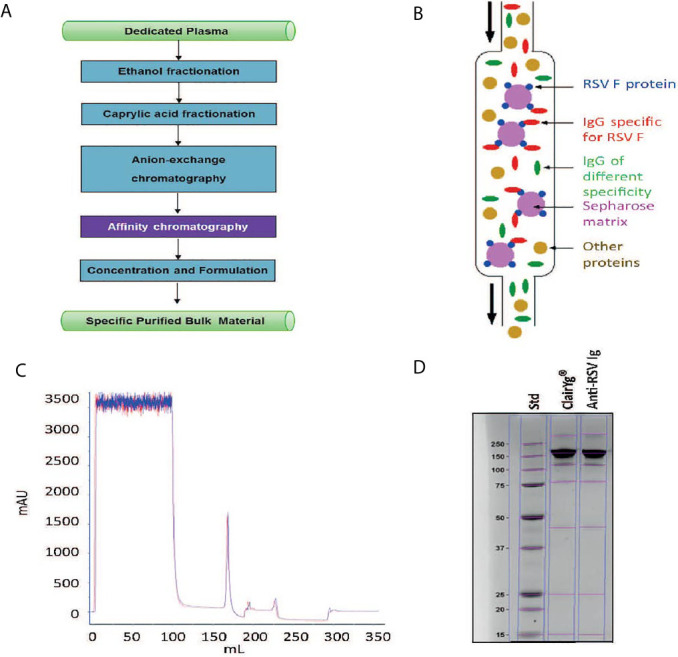
Hyper-enriched anti-RSV Ig manufacturing. **(A)** General approach for anti-RSV Ig manufacturing. **(B)** Principle of the affinity step. **(C)** Hyper-enriched anti-RSV Ig affinity step chromatogram. This figure illustrates the reproducibility of the affinity step with the chromatogram superposition of two independent batches. **(D)** The integrity of the hyper-enriched anti-RSV Ig was compared to a commercial normal polyclonal IgG (ClariYg ^®^) by Coomassie blue staining SDS-PAGE.

The general approach for hyper-enriched anti-RSV IgG (anti-RSV Ig) manufacturing is presented in **Figure 1A**. An intermediate product was obtained from normal plasma after ethanol and caprylic fractionation, a depth filtration and an activated carbon absorption followed by an anion chromatography step. This intermediate product, mainly composed of IgG, was then applied onto an F-RSV affinity sorbent (purple step) to effectively enrich the proportion of anti-RSV Ab. To generate the affinity sorbent, the extracellular domain of the RSV F protein (Met1-Thr529 fused with a polyhistidine tag at the C-terminus, purchased from Sino Biological Inc) was grafted on an NHS-activated Sepharose gel (**Figure 1B**). The starting IgG solution was directly applied on the affinity sorbent using optimized condition for adsorption, wash and elution. The best protocol selected among 13 absorption and 9 elution conditions was the one displaying the higher yield. A final step of concentration and formulation including a filtration step to remove aggregates and endotoxin was performed. The final product recovered, a hyper-enriched anti-RSV Ig fraction, was thus suitable for *in vitro* and *in vivo* non-clinical tests. The purification process, including the affinity step, showed a high reproducibility (see **Figure 1C** for affinity step chromatogram of two batches). Finally, the product was characterized by SDS PAGE (**Figure 1D**). The SDS-PAGE data suggested that anti-RSV Ig purity and integrity was highly similar to what is observed for a commercial normal polyclonal immunoglobulin (ClairYg^®^) and notably that the affinity step did not induce any significant aggregation. Thus, several batches of hyper-enriched anti-RSV Ig were manufactured (two of six different batches are represented here, **Table 1**). By MSD multi-array assay, the hyper-enriched anti-RSV Ig solution showed a level of purity of approximately 15-20% of antibodies specific to F-RSV protein in comparison with less than 0.1% in the initial solution (**Table 1**). Therefore, this cracking process lead to an elution fraction with an enrichment of IgG specific to F-RSV protein of at least 163-fold, as compared to the starting material (**Table 1**).

**Table 1:** The enrichment and the purity of IgG specific to F-RSV protein.

	Ig enrichissment (fold)	purity (%)
Starting material	N. A.	0.09
Elution fraction 1	200	18.01
Elution fraction 2	163	14.67

**Figure 2:**
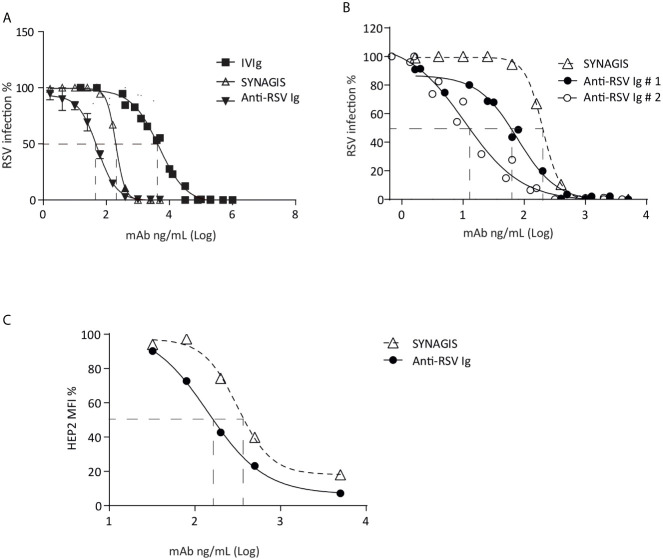
Hyper-enriched anti-RSV Ig characterization. **(A, B)** The biological activity of anti-RSV Ig products was measured by *in vitro* neutralization test assessed on HEp-2 cells and using recombinant RSV-cherry. **(A)** The antiviral activity of the hyper-enriched anti-RSV Ig solution was compared to the starting solution (IVIg) and the antibody control, Synagis^®^ **. (B)** The neutralizing capacity of two batches of hyper-enriched anti-RSV Ig was compared to Synagis^®^. Data are representative of 2 independent assays. **(C)** To determine the ability of the anti-RSV Ig product to neutralize a clinical isolate of RSV, the neutralization assay was performed by flow cytometry on HEp-2 infected cells after immunodetection of RSV-F protein. Results were expressed in MFI, 100 arbitrarily being the MFI obtained with RSV-infected HEp-2 cells without incubation with anti-RSV antibodies. Data are representative of 2 independent assays.

Then, the antiviral ability of the hyper-enriched anti-RSV Ig solution was compared to the initial solution (IVIg). The neutralizing activity of hyper-enriched anti-RSV Ig was measured by the ability to inhibit human RSV infection of HEp-2 cells. These hyper-enriched anti-RSV Ig displayed stronger *in vitro* neutralization effect than IVIg solution (**Figure 2A**). Two batches of the hyper-enriched product were subsequently compared with the marketed monoclonal antibody Synagis^®^. The results of neutralizing test done *in vitro* with a recombinant RSV-cherry (**Figure 2B**) or a clinical strain (**Figure 2C**) revealed a stronger biological inhibitor activity of hyper-enriched anti-RSV Ig than Synagis^®^ antibodies. Thus, the 50% inhibitory concentrations (IC_50_) of two different preparations of hyper-enriched anti-RSV Ig, were at least 3-fold lower than that of Synagis^®^.

### Inhibition of RSV Replication in the Lungs of Mice Treated With Hyper-Enriched Anti-RSV Ig

**Figure 3:**
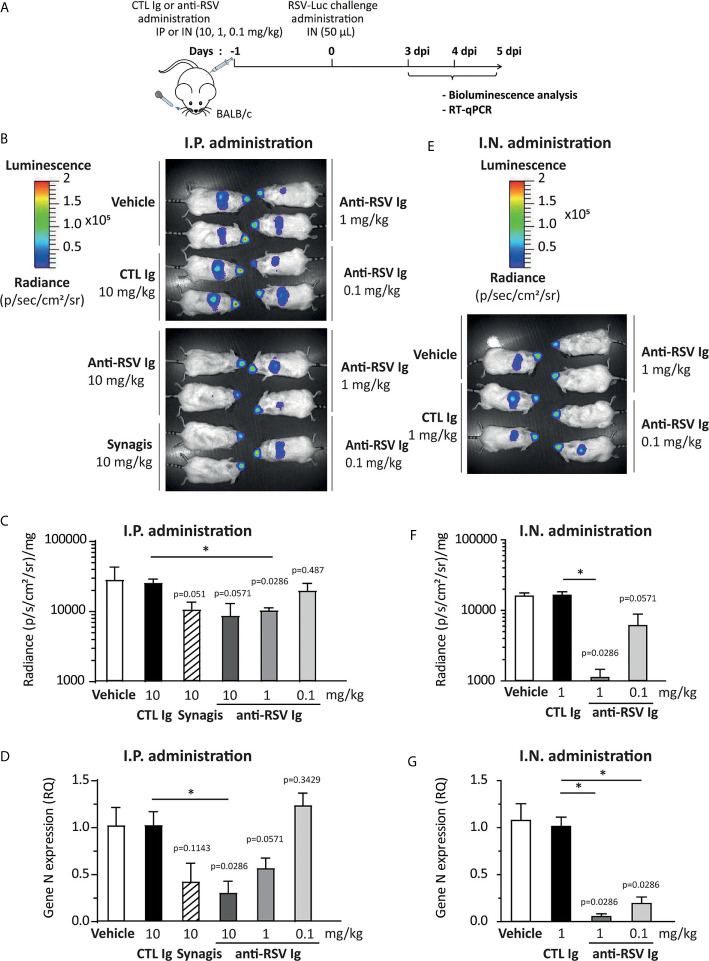
Evaluation of the protective efficacy of anti-RSV Ig in mouse infection model. **(A)** Experimental design showing the time and mode of RSV Ig delivery as well as the time of infection with RSV-Luc and the follow up of virus replication. **(B–D)** BALB/c mice received I.P. administration of 0.1, 1 or 10 mg/kg of anti-RSV Ig or 10 mg/kg of Synagis® before intranasal infection with RSV-luciferase (7 x 10^5^ pfu, 50 µL). As a control of protection/infection, mice were respectively treated with CTL Ig (10 mg/kg) or vehicle. **(E–G)** BALB/c mice received I.N. administration of 0.1 or 1 mg/kg of anti-RSV Ig (50 µL) before intranasal infection with RSV-luciferase (7 x 10^5^ pfu, 50 µL). As a control of protection/infection, mice were respectively treated with CTL Ig (1 mg/kg) or vehicle. **(B, E)** *In vivo* bioluminescence intensity was evaluated at 3, 4 and 5 d.p.i. with IVIS imaging system and is shown at 4 d.p.i. in this figure (dorsal view detection). Depicted images are representative of 2 independent pictures. **(C, F)** *In vitro* luciferase activity was measured in lung homogenates by quantification of bioluminescence emission (radiance in photon/sec/cm^2^/sr) using “Living Image” software after addition of D-luciferin to the lysates, and was normalized to the organ weight. Data are mean ± SEM from *n* = 4 mice. **(D, G)** mRNA level of *RSV N* gene was evaluated by real time RT-PCR in the lungs. Data are mean ± SEM from *n* = 4 mice. Significance is represented on graphs: *P < 0.05.

In order to assess the efficacy of anti-RSV Ig in inhibiting the replication of RSV in an experimental mouse model, we compared two modes of delivery of antibodies, administrated 24 h before viral infection: intraperitoneal (I.P.) *versus* intranasal (I.N.) with different product concentrations (see **Figure 3A** for the experimental design). As controls, mice were treated with irrelevant antibodies (10 mg/kg, CTL Ig) or vehicle (ClairYg^®^ formulation buffer). Mice were then infected with a luciferase-expressing recombinant RSV (RSV-Luc) (20), and viral replication was simultaneously monitored in the nasal turbinates (NT) and in the lungs by *in vivo* bioluminescence. As previously described (20), the peak of replication in the lungs was observed at 4 d.p.i. (data not shown). The replication of RSV-Luc in living mice was detected in the nose and in the lungs of all animals treated with CTL Ig or vehicle (control groups, **Figure 3B**, depicted images of 2 mice are representative of 4 animals). Intra-peritoneal administration of anti-RSV Ig (1 or 10 mg/kg) or Synagis ® resulted in a mild reduction of luciferase activities in the lungs at 4 d.p.i. as compared to the control groups (**Figure 3B**). Interestingly, the inhibition of viral replication in the lungs was dependent upon the dose of anti-RSV Ig as demonstrated by quantification of luciferase activity in the lung lysates at 4 d.p.i. (**Figure 3C**) and by evaluation of viral N gene expression by quantitative RT-PCR performed from lung RNA at 4 d.p.i. (**Figure 3D**). Interestingly, at the same dose (10 mg/kg), the inhibition of viral replication by preventive administration of anti-RSV Ig was comparable to that conferred by Synagis ® although not significantly different from the control groups (**Figures 3C, D**). Similar observations were made at 3 and 5 d.p.i. (data not shown). It should be noted that the delivery of anti-RSV Ig by I.P. route allowed a diffusion of antibodies into the respiratory tract since there was moderate protection against RSV infection in the lungs.

Nasal delivery of anti-RSV Ig was then tested as a way to increase the amount of antibodies in the respiratory tract. When mice received anti-RSV Ig I.N. in 50 μL, luciferase expression monitored in the mouse NT was not affected whatever the dose administered. However, all animals were fully protected against RSV replication in the lungs at the dose 1 mg/kg and some, but not all, were protected at the dose 0.1 mg/kg of anti-RSV Ig (**Figure 3E**, depicted images of 2 mice are representative of 4 animals). These results were further confirmed by the measurement of luciferase activity in the lung lysates (**Figure 3F**) and by evaluation of the viral N gene expression by quantitative RT-PCR (**Figure 3G**). Similar observations were made at 3 and 5 d.p.i. (data not shown). Altogether, these data demonstrate that I.N. administration of anti-RSV Ig successfully prevented viral infection in the lower respiratory tract of mice.

### Preventive Hyper-Enriched Anti-RSV Ig Administration Limited RSV Replication in an Upper Respiratory Tract Model of Infection

**Figure 4:**
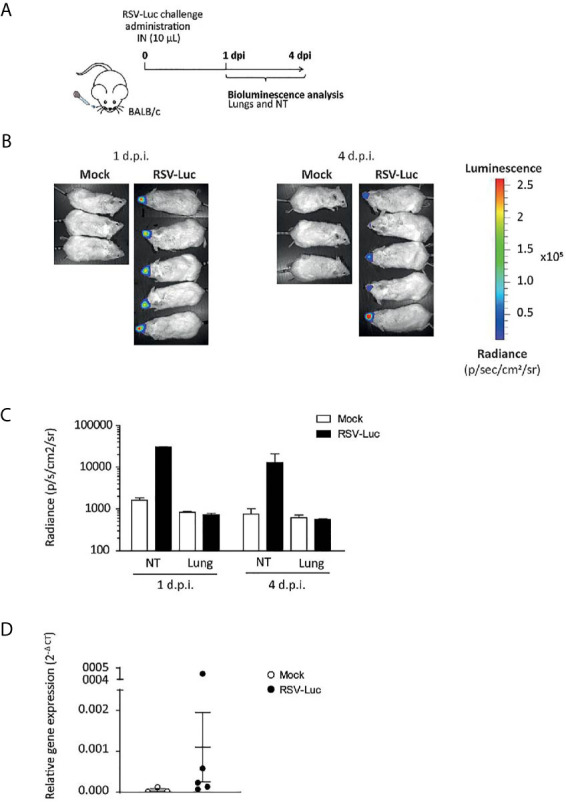
The RSV infection model of the upper respiratory tract. **(A)** BALB/c mice received intranasally a small inoculum volume (10 µL) of RSV-Luc (1.4 × 10^5^ pfu/mice) or mock (supernatant of Hep2 cells) to restrain viral replication to the upper respiratory tract. **(B)** Bioluminescence was measured at 1 and 4 d.p.i. by intranasal injection of 50 μl of D-luciferin and the capture of photon emission (dorsal view) from the whole animal using the IVIS system. **(C)** Luciferase activity was measured in NT or lungs homogenates by quantification of bioluminescence emission (radiance in photon/sec/cm^2^/sr) using “Living Image” software after addition of D-luciferin in the lysates. **(D)** mRNA level of *RSV N* gene was evaluated by real time RT-PCR in the lungs and expressed with the formula (2^-ΔCt^). Data are mean ± SEM from *n* = 3 mock-infected mice or *n* = 5 RSV-Luc-infected mice.

The volume of the I.N. inoculum is critical for the diffusion of the virus in the nasopharynx and lungs. Indeed, it has been previously shown that reduction of the inoculum volume to 20 μL in mice resulted in a larger retention in the nasopharynx (21). Therefore, we adapted a model of upper respiratory tract infection consisting in the I.N. administration of 10 μL of RSV in mice. **Figure 4A** illustrates the replication of RSV-Luc in mice after nasal inoculation of the virus. Viral replication in NT by the measurement of luciferase activity was evident in RSV-infected mice as compared to the control groups at 1 d.p.i. and at 4 d.p.i. (**Figures 4B, C**). Of note, viral replication measured by the luciferase activity (**Figures 4B, C**) and the viral N gene expression by quantitative RT-PCR (**Figure 4D**) was almost undetectable in the lungs.

**Figure 5:**
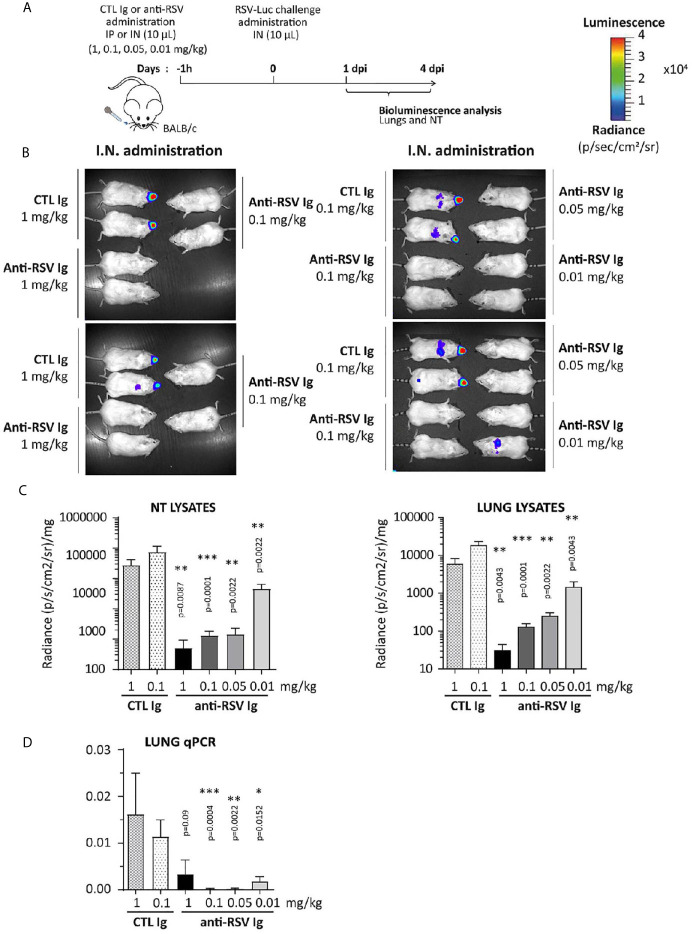
Efficacy of anti-RSV Ig intranasal administration at low doses on upper respiratory tract RSV infection in mice. **(A)** BALB/c mice received I.N. administration of 0.01 to 1 mg/kg of anti-RSV Ig in 10 µL one hour before I.N. injection of RSV-luciferase (1.4 x 10^5^ pfu, 10 µL). As a control of RSV-Ig, mice were treated with CTL Ig (0,1 or 1 mg/kg). **(B)** *In vivo* bioluminescence intensity was evaluated at 4 d.p.i. with IVIS imaging system (dorsal view detection). Depicted images are representative of 3 independent pictures. **(C)** Luciferase activity was measured in NT and lung homogenates by quantification of bioluminescence emission (radiance in photon/sec/cm^2^/sr) using “Living Image” software after addition of luciferin in the lysates, and was normalized to the organ weight. **(D)** mRNA level of *RSV N* gene was evaluated by real time RT-PCR in the lungs and expressed with the formula (2^-ΔCt^). Data are mean ± SEM from *n* = 6 mice for CTL Ig-treated mice or all doses of anti-RSV Ig and *n* = 12 for mice treated with 0.1 mg/kg of anti-RSV Ig. Significance is represented on graphs: *P < 0.05; **P < 0.01; ***P < 0.001.

The ability of anti-RSV Ig to inhibit RSV replication was further evaluated in this upper respiratory tract infection model. One hour before RSV infection, the anti-RSV Ig (0.01 to 1 mg/kg) were instilled intranasally in a small volume (10 µL) to limit the distribution of antibodies within the NT (see **Figure 5A** for the experimental design). Upon RSV infection, the bioluminescence intensity in living mice increased in the NT of mice treated with CTL Ig (control group) at 1 d.p.i. (data not shown) and 4 d.p.i., confirming that viral replication occurred in the NT (**Figure 5B**, depicted images of 4 mice are representative of 6 animals). In contrast, all animals treated with anti-RSV Ig showed no signal in the NT at all administered dose. Thus, nasal administration of low doses of anti-RSV Ig conferred significant protection against RSV replication in the upper respiratory tract, as confirmed by quantification of bioluminescence in NT lysates at 4 d.p.i. (**Figure 5C**, **Supplemental Figure S1** for 1 d.p.i., no significant difference between control groups treated with 0.1 mg/kg or 1 mg/kg).

These experimental conditions were designed to restrict the infection to the NT. However, at 4 d.p.i, a bioluminescence signal could be observed in the lungs of CTL-treated individuals and of a few mice treated with the lowest concentration of anti-RSV Ig (0.01 mg/kg) (**Figure 5B**). Luminescence signals measured in lung lysates of CTL animals were lower by one log than those in NT lysates (**Figure 5C** and **Supplemental Figure 1** for 1 d.p.i), and were significantly reduced in all groups of mice treated with anti-RSV Ig compared to controls. The quantification of the N viral gene by RT-qPCR confirmed the very low replication of RSV in the lungs at 1 d.p.i. (**Supplemental Figure 1C**) and at 4 d.p.i. (**Figure 5D**) in anti-RSV Ig-treated mice. Overall, these results indicate that I.N. administration of low doses of anti-RSV Ig provided nearly complete protection against RSV replication in the upper respiratory tract in mice.

**Figure 6:**
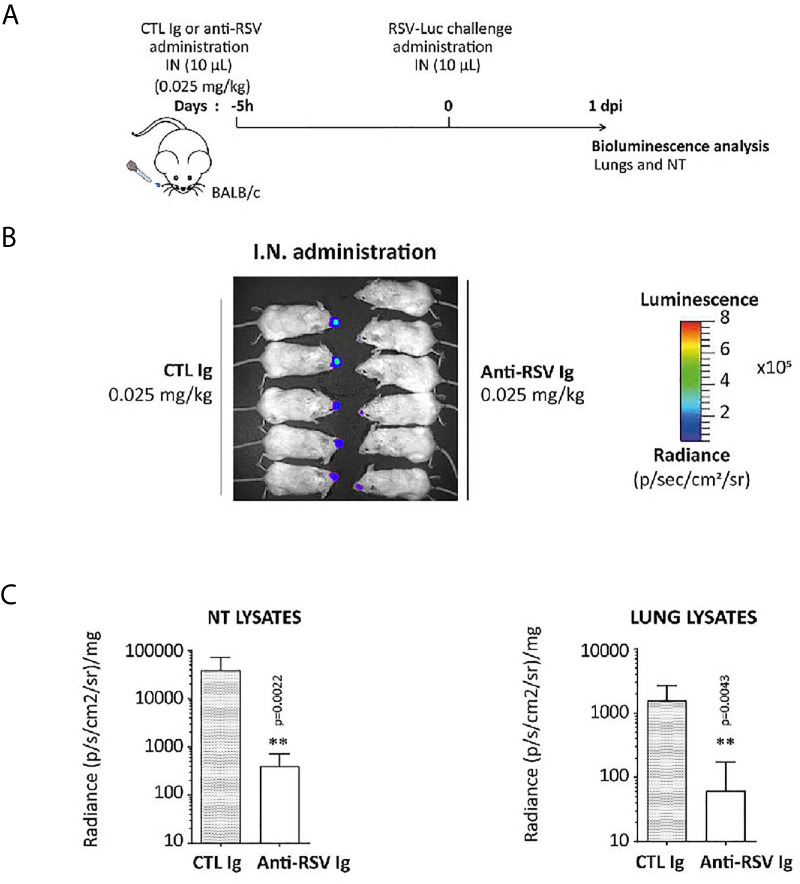
Preventive effect of anti-RSV Ig intranasal administration on upper respiratory tract RSV infection in mice. **(A)** BALB/c mice received I.N. administration of 0.025 mg/kg of anti-RSV Ig in 10 µL five hours before I.N. injection of RSV-luciferase (1.4 x 10^5^ pfu, 10 µL). As a control of infection, mice were treated with CTL Ig. **(B)** *In vivo* bioluminescence intensity was evaluated at 1 d.p.i. with IVIS imaging system (dorsal view detection). **(C)** Luciferase activity was measured in NT and lung homogenates by quantification of bioluminescence emission (radiance in photon/sec/cm^2^/sr) using “Living Image” software after addition of luciferin in the wells, and was normalized to the organ weight. Data are mean ± SEM from *n* = 6 mice for CTL Ig-treated mice and *n* = 5 for mice treated with 0.025 mg/kg of anti-RSV Ig. Significance is represented on graphs: **P < 0.01.

Finally, the ability of anti-RSV Ig nasal treatment to reduce viral replication was tested with a very low dose of anti-RSV Ig (0.025 mg/Kg) administrated five hours before the viral challenge (see **Figure 6A** for the experimental design) in order to investigate a preventive antibody instillation several hours before viral infection. Virus replication was monitored by bioluminescence emission in living mice at 1 d.p.i, the optimal time for viral replication in the nasal cavity (**Figure 6B**). The replication of RSV-Luc in living mice was detected mainly in the NT but not in the lungs of mice treated with irrelevant Ig (control group), as expected with this RSV infection model at 1 d.p.i. In contrast, all animals treated with anti-RSV Ig showed no or very weak signal in the NT (**Figure 6B**), confirmed by quantification of bioluminescence in NT lysates at 1 d.p.i. (**Figure 6C**). Thus, this experiment showed that the prophylactic instillation of anti-RSV Ig, even several hours before challenge and at a very low dose, provided a significant reduction of RSV replication in the upper respiratory tract in mice.

## Discussion

RSV is a major cause of acute lower respiratory tract infection for all fragile populations including young children, immunocompromised adults, patients with chronic respiratory affections and the elderly. The only intervention licensed today is Synagis^®^, a humanized monoclonal antibody administered by the intramuscular route and with a very restricted indication to a specific infant segment. So far, none of the vaccine strategies under clinical trials has reached the market. However, immunity acquired after natural infection appears very effective since RSV pathogenicity is mild to asymptomatic for the healthy adult population. Thus, passive immunization aiming to transfer some Ig protection from healthy people to fragile patients appears logical. Nevertheless, this approach remains limited by the low Ig titers obtained after purification from strongly immunized donors, the so-called hyper-immune Ig molecule class (1).

Nowadays, human plasma derived immunoglobulins are used as replacement therapies in primary immune deficiency diseases (PIDD) or secondary immuno-deficiencies (SID) or in the treatment of autoimmune diseases (AIDs). The consumption of IVIg represents roughly 30 and 70% in these indications, respectively. Although the molecular mechanisms by which IVIg act in these two groups of indications are different (in a very simplified way, Fab fragment mainly for epitope-specific anti-infective activity and Fc fragment for immunomodulation *via* FcR), the administered products are often the same. In order to rationalize their use and increase the available quantities for patients, attempts have been made in the past to divide IVIg into Fab and Fc fractions. These attempts remain however very limited, despite encouraging clinical results (22, 23).

Here, we devised a cracking solution for IVIg, as done for the oil refinery. The driving idea was to extract from the same source of PdIgs (Plasma or semi purified fractions) different pathogen-specific products meeting unsatisfied medical needs. Therefore, instead of addressing the need of one patient, each liter of plasma will serve for the treatment of several patients, allowing an optimized use of this limited resource. The main difference compared with oil cracking is that the cracking of IVIg is based on a cascade of affinity chromatography using viral antigens as ligands, derived from the targeted viruses, such as RSV, as in the present study.

Using this concept, we have succeeded in extracting from the same source of material (ClairYg^®^,a commercial IVIg preparation) 4 candidate products (anti-RSV supposedly from symptom-free to mild cold in healthy immunocompetent adults, anti-tetanus and anti-hepatitis B hyper enriched Igs from vaccinated adults and one fraction which could be used in the treatment of AIDs). As a proof of concept for the IVIg cracking concept, the details of the anti-RSV product are reported in this study. Using an optimized F-RSV affinity chromatography, a hyper-enriched anti-RSV Ig solution was extracted from ClairYg^®^ preparation. The purification step was reproducible and resulted in an enrichment of anti-RSV Ig by at least 160-fold, as determined by MSD multi-array assay. The antiviral activity of the purified product was first assessed *in vitro* by measuring its ability to inhibit RSV infection of HEp-2 cells. These hyper-enriched anti-RSV Ig displayed a strong *in vitro* neutralization effect on different RSV strains. Noteworthy this effect was stronger than Synagis^®^. Considering that the total anti-RSV IgG content of hyper-enriched anti-RSV Ig solution is expected to be at least 15-20% (see **Table 1**), a higher activity could be reached by an improvement of the enrichment step. Moreover, the I.P. or I.N. (50 µL) pre-administration of anti-RSV Ig solutions was able to limit RSV replication in the lungs of treated-mice, demonstrating that these hyper-enriched antibodies provide a successful inhibition of RSV replication in the lower respiratory tract. Chadock’s group previously demonstrated that the therapeutic effect of IVIG was dependent on their mode of administration in different strategies for experimental immunotherapy of RSV infection in cotton rats or owl monkeys (24, 25). Furthermore, a small volume (< 20 μL) of inoculum administered intranasally resulted in a retention of products in the nasopharynx and in a limited distribution in the lungs (21). Therefore we evaluated the interest of combining the high potency of this product with an intranasal instillation of a small volume (10 µL) to enhance the nasopharynx distribution of anti-RSV Ig, and to prevent the early step of RSV infection in the upper respiratory tract of mice. Thus, the neutralizing activity combined with a very local administration in the NT led to a nearly complete protection against RSV infection in a relevant *in vivo* model. Altogether the results reported here clearly demonstrate the interest of the approach we propose, since it allowed an almost complete protection against RSV infection that could not be reached with Synagis^®^ regardless the doses parenterally given. In addition to their better protection efficacy, anti-RSV-Ig are expected to generate cost-savings as compared with Synagis^®^. Owing to the intramuscular administration route of Synagis^®^ the annual treatment is approximately 5.3 g per year for an adult of 70 kg of body weight. Using public non discounted price this corresponds to approximately $ 165 000. The nasal route of administration will allow using much less product per patient and per year (from our current results, annual treatment product quantities are estimated at less that 100 mg/patient/year). Additional savings are expected from the manufacturing process since the plasma starting material and facility cost will be shared with other products (for example, this costing sharing structure allows the plasma fractionation industry to sell for example human IVIG at cost ranging from $ 30 to $ 70 per g which is 10 to 20 folds less than average price of monoclonal antibodies). Finally higher potency, resulting from polyclonal nature of our anti-RSV antibodies is also expected to contribute to the global cost saving. Moreover, we believe that the local administration of Ig requires less product amounts per patient compared to the parenteral route, hence leading to treatment cost savings. The preventive instillation of anti-RSV Igs, even at very low doses, provided a significant reduction of RSV infection in the upper respiratory tract in mice. It will be interesting to associate a mucoadhesive buffer with hyper-enriched anti-RSV Ig in order to enhance the interaction with the mucus layer covering the mucosal epithelial surface and to extend the duration of stay of inhaled products in the NT (26). Thus, the combination of a local administration and low product amount allowed a level of protection never reached before with very low dose of hyper-enriched anti-RSV Ig and suggested a novel preventive treatment that should protect against RSV infections with a very small quantity of product administered daily (or twice daily) during hospitalization (i.e. after graft or any treatment or condition leading to immunocompromised situation) or during all the RSV season.

It is noteworthy that the anti-RSV Ig used for our proof of concept study were obtained using the extracellular domain of F as an affinity bait. This extracellular version of F contains the F1 and F2 subunits, presumably mainly in the post-fusion conformation, since it is known for its better stability (16). It is likely that a fraction of anti-RSV neutralizing antibodies were left out in the enrichment process, namely the neutralizing antibodies specific for preF (19). The efficacy of our anti-RSV Ig could be improved by using the stabilized preF DS-Cav1 as an affinity ligand (17). An even better approach would be to use epitope-scaffold mimicking the 3D-structure of potent neutralizing F-RSV epitopes (27, 28). This latest strategy would ascertain to eliminate from the anti-RSV-Igs, the non-neutralizing antibodies possibly mediating disease enhancement (29).

In summary, we report a prophylactic strategy based on the generation of a new class of hyper-enriched IgG purified by a relevant affinity enrichment of normal plasma Igs against F-RSV protein. Immunoglobulins from symptom-free- to convalescent adults exposed to RSV may be an even better started source for affinity enrichment. Altogether, these data suggest that this approach is very promising for further development to protect patients at risk of developing severe RSV disease. Moreover, the general hyper-enriched Ig concept should provide solutions to treat and prevent several other infectious diseases in fragile populations especially in the absence of vaccination. In addition, by protecting the upper respiratory tract, this approach is expected to better limit the virus transmission than any passive or active immunization achieved with systemic administration. This is particularly relevant to break the chain of transmission of respiratory infectious agents. This innovative bioprocess of Ig cracking from either normal plasma or from convalescent plasma constitutes a novel technological strategy to develop new tools to control emerging viral diseases, such as COVID-19.

## Data Availability Statement

The original contributions presented in the study are included in the article/**Supplementary Material**. Further inquiries can be directed to the corresponding authors.

## Ethics Statement

The animal study was reviewed and approved by COMETHEA (INRAE and AgroParisTech). All experiments were approved with authorization number 15-50.

## Author Contributions

EJ, DD, GP, and SC conceived the project, designed and performed experiments, contributed to execution and analysis of the described study. EJ, DD, SC, and SR wrote the manuscript. PM, CR, SR, J-FE, CR, and M-AR-W helped with discussion related to conceptualization of the study and designed experiments.

MM, SM, and LB contributed to the purification and the *in vitro* analysis of the hyper-enriched Ig. EJ, DD, CC, DL, CF, and CD performed *in vivo* infection and *in vivo* imaging studies and analysis. M-AR-W and J-FE engineered the RSV-Luc and RSV-mCherry used for *in vivo* experiments. All authors contributed to the article and approved the submitted version.

## Conflict of Interest

The authors declare that the research was conducted in the absence of any commercial or financial relationships that could be construed as a potential conflict of interest.

## Supplementary Material

Supplementary Figure 1 Evaluation of anti-RSV Ig efficacy at low doses on upper respiratory tract RSV infection at 1 d.p.i. BALB/c mice received I.N. administration of 0.01 to 1 mg/kg of anti-RSV Ig in 10 µL one hour before I.N. injection of RSV-luciferase (1.4 x 10^5^ pfu, 10 µL). As a control of RSV-Ig, mice were treated with CTL Ig (0,1 or 1 mg/kg). **(A, B)** Luciferase activity was evaluated at 1 d.p.i. with IVIS imaging system in NT **(A)** and lung homogenates **(B)** by quantification of bioluminescence emission (radiance in photon/sec/cm^2^/sr) using “Living Image” software after addition of luciferin in the lysates, and was normalized to the organ weight. **(C)** mRNA level of *RSV N* gene was evaluated by real time RT-PCR in the lungs and expressed with the formula (2^-ΔCt^). Data are mean ± SEM from *n* = 6 mice for CTL Ig-treated mice or all doses of anti-RSV Ig and *n* = 12 for mice treated with 0.1 mg/kg of anti-RSV Ig.
