# Supplementary figures and images for: Hyper-Enriched Anti-RSV Immunoglobulins Nasally Administered: A Promising Approach for Respiratory Syncytial Virus Prophylaxis
Source: Front Immunol. 2021 Jun 7;12:683902. doi: 10.3389/fimmu.2021.683902 (PMC8215542; doi:10.3389/fimmu.2021.683902)

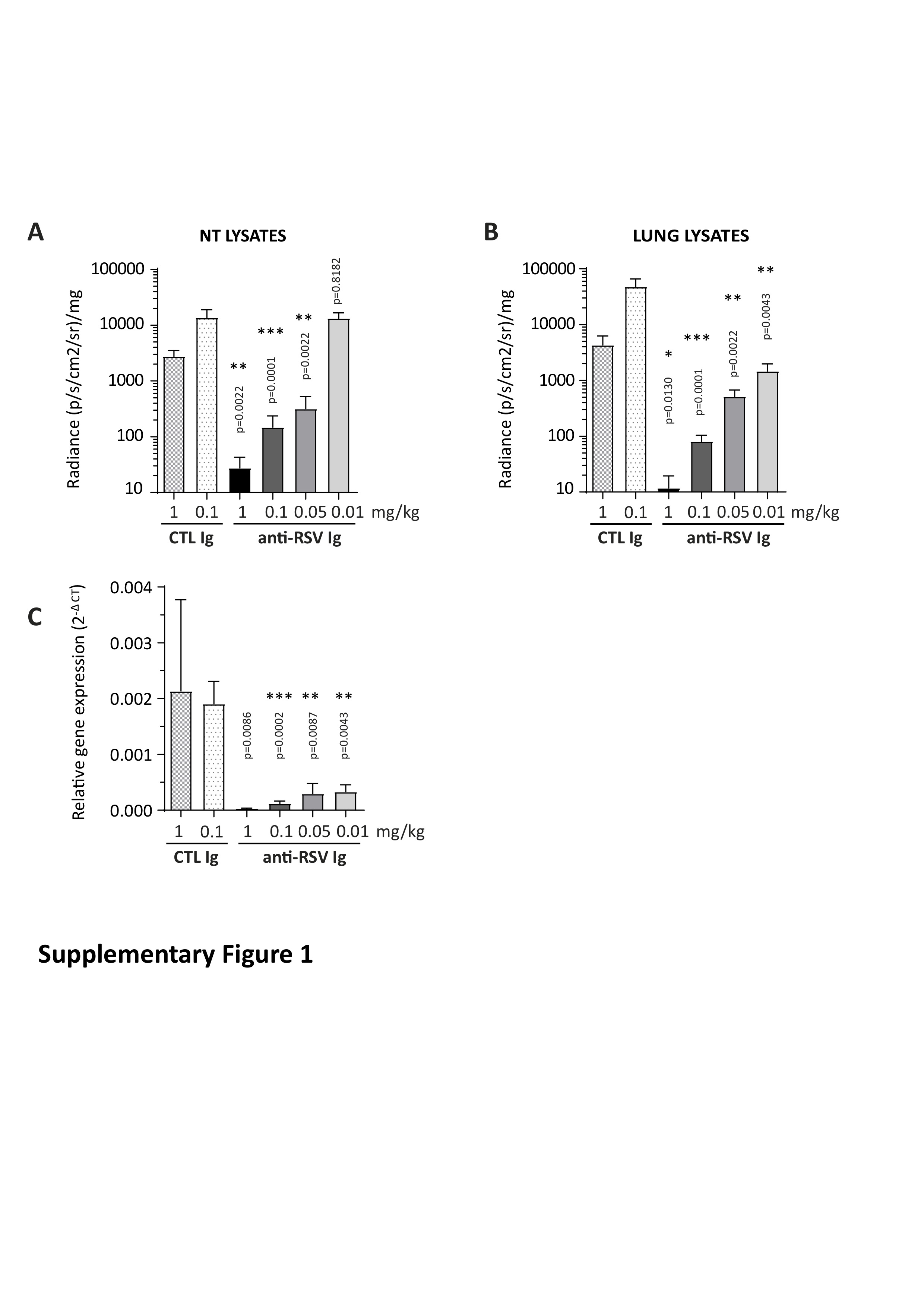

Supplement: Supplementary Figure 1 — Evaluation of anti-RSV Ig efficacy at low doses on upper respiratory tract RSV infection at 1 d.p.i. BALB/c mice received I.N. administration of 0.01 to 1 mg/kg of anti-RSV Ig in 10 µL one hour before I.N. injection of RSV-luciferase (1.4 x 10^5^ pfu, 10 µL). As a control of RSV-Ig, mice were treated with CTL Ig (0,1 or 1 mg/kg). (A, B) Luciferase activity was evaluated at 1 d.p.i. with IVIS imaging system in NT (A) and lung homogenates (B) by quantification of bioluminescence emission (radiance in photon/sec/cm2/sr) using “Living Image” software after addition of luciferin in the lysates, and was normalized to the organ weight. (C) mRNA level of RSV N gene was evaluated by real time RT-PCR in the lungs and expressed with the formula (2-ΔCt). Data are mean ± SEM from n = 6 mice for CTL Ig-treated mice or all doses of anti-RSV Ig and n = 12 for mice treated with 0.1 mg/kg of anti-RSV Ig. [file Image_1.jpeg]
